# A bibliometrics review of the journal *mindfulness*: science mapping the literature from 2012 to 2022

**DOI:** 10.3389/fpsyg.2024.1378143

**Published:** 2024-05-17

**Authors:** Chuan-Chung Hsieh, Shun Li

**Affiliations:** Department of Education and Learning Technology, National Tsing Hua University, Hsinchu, Taiwan

**Keywords:** mindfulness, science mapping, bibliometric analysis, knowledge graph, visualization analysis

## Abstract

This study conducts a bibliometric analysis using the Web of Science database on 1,950 articles published in the journal *Mindfulness* from 2012 to 2022. By constructing a knowledge graph, the research delineates the evolution, stages of development, and emerging trends in the field of mindfulness. Significant growth in the annual publication volume has been observed since 2012, with the research progression segmented into three distinct phases. The United States has emerged as a pivotal contributor to the field, dominating in terms of publication volume, researcher involvement, and institutional contributions. Through the application of keyword co-occurrence and reference co-citation analysis, five principal clusters were identified, focusing on mindfulness, meditation, depression, stress, and self-compassion, underscoring these as focal research areas. Furthermore, the exploration of mindfulness within the educational sphere in Taiwan is still nascent, signaling a critical need for bolstered research support in diverse thematic domains.

## Introduction

Mental health, a cornerstone of contemporary society, substantially influences national productivity and interpersonal relationships. Recent advances in mindfulness research suggest that cultivating mindfulness fosters positive and resilient attitudes towards evolving social contexts. Kabat-Zinn’s pioneering work in 1979 integrated Buddhist mindfulness meditation into the medical realm, inaugurating Mindfulness-Based Stress Reduction (MBSR) clinics—a milestone in healthcare applications of mindfulness (Wen, 2016).

As research into mindfulness has deepened, its applications have broadened from medicine to fields such as psychology, education, business, and even commercialization (Wilson, 2014). Empirical studies indicate that mindfulness enhances self-acceptance, care, and courage, which in turn uplifts the quality of life (Dobkin and Zhao, 2011). Additionally, it mitigates symptoms of stress, depression, and anxiety, bolsters positive emotions, and improves psychological health (Keng et al., 2011; Lakhan and Schofield, 2013; Penman and Burch, 2013). It also positively impacts attention and emotional regulation (Wen, 2013), and strengthens interpersonal relationships (Grossman et al., 2004; Davis and Hayes, 2011). As a holistic approach to psychological and physical well-being, mindfulness education encourages students to deepen their reflective thinking, enhance awareness, and apply mindfulness practices, thus continually advancing their academic and health outcomes. For instance, Napoli et al. (2005) observed that mindfulness improves children’s selective attention, mental health, and cognitive functions, enhancing overall well-being. Mindfulness interventions have also been shown to mitigate depressive symptoms in adolescents (Raes et al., 2014; Kallapiran et al., 2015) and to enhance cognition, academic performance, behavior, and socio-emotional qualities among primary and secondary students (Maynard et al., 2017). Hsieh (2018) advocates for the integration of mindfulness into school education and management, aiming to foster a comprehensive understanding of life’s significance, the pursuit of meaningful values, and the promotion of care and social responsibility.

In Taiwan, mindfulness research, albeit more recent, focuses on enhancing attention, body and mind awareness, emotional processing, and stress regulation through mindfulness practices, or explores its benefits in physical and mental health, professional development, and patient care (Chen et al., 2019). Thus, it is necessary for Taiwan to further expand the application domains of mindfulness research and to support its development through government policies, as well as to strengthen interdisciplinary collaborations to deepen the understanding of mindfulness effects across various groups. For example, Jin and Liu (2017) implemented targeted mindfulness interventions for special student groups, providing insightful experiences applicable to broader student populations; Jiang et al. (2022) integrated insights from psychology, education, and sociology to explore how mindfulness parenting positively affects parent–child relationships and alleviates behavioral issues in children, contributing significantly to societal welfare.

The advent of Knowledge Graph technology has revolutionized the exploration of disciplines, academic communities, and intellectual traditions through the analysis of journal articles. Unlike traditional reviews and meta-analyses, bibliometric analysis offers a detailed summary of a field’s literature metrics and knowledge structure by examining the structural relationships among authors, countries, institutions, and themes, employing statistical methods such as article counts, reference co-citation analysis, and impact factors (Donthu et al., 2021). This approach provides a more nuanced understanding of the dynamics across various scientific fields, enhancing the scope and depth of academic exploration.

To date, the systematic construction of knowledge graphs in the realm of mindfulness research remains limited. The journal *Mindfulness* serves as a critical resource for advancing the assessment, prevention, treatment, counseling, training, and collaboration of mindfulness theories and interdisciplinary studies. Given this backdrop, a comprehensive analysis of the mindfulness research literature is essential. This analysis will facilitate a macroscopic understanding of the developmental trajectory, knowledge base, research hotspots, and future research directions in this field. Moreover, it will inform recommendations and enhancements for education in Taiwan.

This paper employs VOSviewer (v.1.6.18) to analyze mindfulness-related research from 2012 to 2022, exploring thematic developments and presenting the findings via a knowledge graph, providing a foundational reference for future studies. This study addresses the following research questions:

1. What is the publication count and growth trajectory of mindfulness literature?
2. Which authors and countries have the most significant influence on mindfulness research?
3. What are the primary research hotspots within the field of mindfulness?
4. What implications does mindfulness research hold for the educational in Taiwan?

### Literature review

“Mindfulness,” often associated with terms such as contemplation, introspection, and concentration, originates from the Buddhist term “sammā-sati,” which translates to “Right Mindfulness” or simply “Mindfulness.” This term encapsulates the concepts of awareness, attention, and remembering, essential for alleviating physical and mental stress (Lv, 2014). Buddha, who lived approximately 2,500 years ago, emphasized that mindfulness is crucial for overcoming ‘attachment, aversion, and delusion.’

The theoretical foundation of mindfulness research was laid by Ellen Langer, a social psychologist at Harvard University. In her 1989 work, she proposed that many negative life outcomes, such as unhappiness, accidents, and poor health, could result from a lack of mindfulness (Langer, 1989). Thus, she viewed mindfulness as both a method of mental training and a way of life, helping individuals to observe changes within their bodies and minds and to maintain an open, accepting, and clear presence in the moment (Lin, 2013).

Mindfulness, deeply rooted in religious traditions, has evolved significantly under their influence. Wen (2013) emphasized that mindfulness focuses on present awareness and mental states, which profoundly impact human physical and mental health. The process involves causal interactions that construct what is termed “experience,” generated through the senses (eye, ear, nose, tongue, body, mind) and integrated conceptually. Mindfulness categorizes the six senses into five aggregates: form (material), sensation, perception (identification and evaluation), volitional formations (responses and actions), and consciousness. The practice asserts that identification with “self,” “mine,” or “myself” is illusory, and true awareness is based on this realization. Buddha taught that mental changes are constant and recognizing this allows for greater flexibility and acceptance in responding to life’s changes (Ronald et al., 2009).

Mindfulness has been extensively researched within medicine, modern psychology, and social psychology, influenced initially by psychologist Kabat-Zinn. In 1979, he introduced the Mindfulness-Based Stress Reduction (MBSR) technique, applying mindfulness to clinical psychology with a focus on emotional regulation, stress management, mind–body interaction, and meditation practices. Numerous studies have confirmed mindfulness’s effectiveness in alleviating physical and psychological distress (Kabat-Zinn, 2003). Recent research indicates positive effects of mindfulness interventions on individuals with amphetamine-type substance use disorders (SUDs), highlighting improved mindful awareness and certain electroencephalographic functional connectivity (Zhang et al., 2019). Additionally, a meta-analysis of 40 randomized controlled trials on mindfulness-based interventions (MBIs) for SUDs, excluding tobacco use disorders, suggests these interventions might slightly reduce substance use days compared to standard care, cognitive-behavioral therapy, or pharmacotherapy, though further research is needed to confirm their overall effectiveness (Goldberg et al., 2021). MBIs have also been successfully applied to a range of addictions, from smoking to alcohol, and behavioral addictions like gambling disorders, reducing dependency, cravings, and improving emotional states. Common MBI methods include Mindfulness-Based Relapse Prevention, Mindfulness Training for Smokers, and Mindfulness-Oriented Recovery Enhancement, with the integration of MBIs with treatment as usual (TAU) or other active treatments proving most effective (Sancho et al., 2018). MBSR courses have not only benefitted the fields of medicine, psychology, and education but have also been widely promoted within the corporate sector, significantly improving physical and mental health, emotions, and quality of life (Hsieh, 2019). Research by Valentine and Sweet (1999) showed that mindfulness meditators exhibit better psychological health than those practicing focused meditation. Various studies have explored the attention mechanisms of mindfulness meditation, correlating it with mental health improvements through attention regulation, body awareness, emotional regulation, and changing self-perceptions. Evidence suggests mindfulness meditation training enhances attention-related behavioral responses, cognitive abilities, reduces stress, and increases well-being (Jha et al., 2007; Chiesa et al., 2011; Hölzel et al., 2011; Eberth and Sedlmeier, 2012; Jensen et al., 2012).

Research on self-compassion, particularly prevalent in Western studies, highlights its components—self-love, reduced self-judgment, decreased feelings of isolation, mindfulness, and lessened over-identification. Self-compassion interventions foster self-care, kindness, and tolerance, aiding individuals, especially the youth, in developing positive internal processing systems and reducing mental health issues. Its core aspects include treating oneself kindly, recognizing common humanity, and maintaining mindfulness (Neff, 2003a; MacBeth and Gumley, 2012; Körner et al., 2015; Costa et al., 2016; Muris et al., 2016; Neff et al., 2017, 2019). Additionally, mindfulness regulates emotions, enhances attention, reduces stress, and positively impacts interpersonal communication and creativity (Grossman et al., 2004; Corcoran et al., 2010; Farb et al., 2010; Davis and Hayes, 2011; Keng et al., 2011; Lakhan and Schofield, 2013; Lawlor, 2014; Penman and Burch, 2013; Wall, 2014; Willis and Dinehart, 2014; Laukkonen et al., 2020).

Compared to Western studies, mindfulness research in Taiwan shows distinct traits. In quantitative studies, there is a strong focus on developing mindfulness scales, therapeutic interventions, and curriculum implementation. For example, Huang et al. (2015) conducted reliability and validity analyses of the “Taiwanese Version of the Five Facet Mindfulness Questionnaire”; Liu and Rau (2015) investigated how mindfulness meditation enhances attention; Yang (2016) integrated mindfulness practices into curricula and assessed impacts through pre- and post-tests using the “Five Facet Mindfulness Questionnaire,” “Stress Perception,” and “Mindfulness Attention Awareness Scale.” In qualitative research, studies often focus on specific benefits or challenges encountered during mindfulness practices. For instance, Zheng et al. (2013) examined the effects of adult mindfulness courses on depression, anxiety, and mindfulness awareness, finding no significant differences; Shin and Jin (2010) discussed how “Zen Mindfulness Groups” influence intern counselors’ self-focus and professional practices. These studies provide insights into the effects of mindfulness on specific target groups and contribute to a deeper understanding of factors influencing mindfulness practices.

The ongoing deepening of mindfulness practice enables scholars to gain profound insights into their behavioral and cognitive patterns, reflecting on and adjusting their values and beliefs. This integration of awareness and action not only advances research in mindfulness but also demonstrates its significant applicative value across various fields such as medicine, psychology, and education, effectively enhancing individual well-being and broader societal impact.

## Method

In recent years, bibliometric analysis has emerged as a fundamental method in scientific research, providing quantitative and statistical evaluation of scholarly outputs such as journal articles, citation counts, and impact factors (Donthu et al., 2021). First introduced by Pritchard in 1969, the concept of bibliometrics pertains to the systematic analysis of scholarly literature to understand the evolution and structural dynamics of academic disciplines (Pritchard, 1969). This review applies bibliometric techniques to scrutinize significant literature and themes within the field of mindfulness research, aiming to delineate the current state of the discipline and project future research directions.

The analysis utilizes VOSviewer (version 1.6.18) as the principal tool, capitalizing on its ability to create knowledge maps that visualize relationships between various bibliometric elements. These include descriptive analysis, examination of authorship and geographical distribution, keyword co-occurrence, and reference co-citation analyses. VOSviewer is renowned for its effectiveness in graphically representing scientific landscapes, thereby facilitating the exploration of connections across diverse research areas (Van Eck and Waltman, 2010; Zupic and Čater, 2015).

Keyword co-occurrence analysis is particularly valuable for detecting research development trends and assessing the status of domains (Zhang, 2013; Yang, 2015). In this analysis, keywords with higher co-occurrence frequencies are indicative of prevailing research hotspots, highlighting the central themes within the field. This method employs visual representations of co-occurrence networks, where nodes represent keywords, encapsulating the cumulative knowledge of a domain, and links illustrate the relationships between word pairs, denoting their co-occurrence (Radhakrishnan et al., 2017).

Reference co-citation analysis is employed to measure the similarity between documents or topics based on the frequency of their co-citations (Small, 1973). The density of connection lines in the co-citation network graphically represents the strength of relationships between documents, providing insights into the interconnectedness of research themes. This type of analysis is crucial for identifying topics that have gained traction in the short term and may also indicate emerging research frontiers (Zhang, 2013)

### Data source, procedure, and analytic software

This study employs data sourced from the Web of Science (WoS) Core Collection, which includes the Social Sciences Citation Index (SSCI), Science Citation Index Expanded (SCI-Expanded), and Arts & Humanities Citation Index (A&HCI). These databases are recognized for their extensive reach and integration across multiple disciplinary areas, holding significant academic influence (Zyoud et al., 2017). WoS is particularly noted for its comprehensive coverage, with approximately 99.11% of its indexed journals also featured in the Scopus database, underscoring its broad applicability and prominence in global research landscapes (Singh et al., 2021). The journal *Mindfulness*, indexed in the SSCI and ranking highly within the Psychiatry and Clinical Psychology categories, consistently achieves Q1 and Q2 status, indicative of its high-quality scholarly output. Thus, the selection of research papers from these sources ensures a reliable representation of the mindfulness research quality, supporting the validity of the study’s findings.

**Figure 1:**
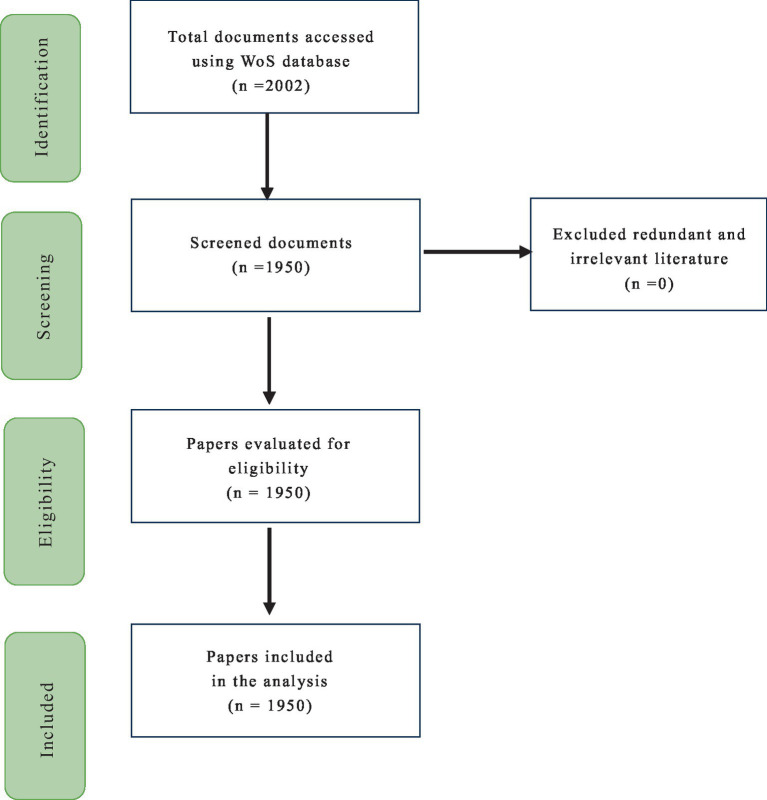
Flow diagram of study selection process.

Bibliometric analysis serves as a crucial tool for elucidating the accumulated scientific knowledge and developmental nuances of established fields through the systematic examination of large volumes of unstructured data (Donthu et al., 2021). This study adopts the PRISMA (Preferred Reporting Items for Systematic Reviews and Meta-Analyses) framework (Figure 1), guiding the systematic literature review process to ensure transparency and standardization in the bibliometric methodology. This approach aids in the precise selection of relevant outcomes (Moher et al., 2009). This study specifically focuses on articles from the *Mindfulness* journal indexed in the WoS database, covering the period from 2012 to 2022. The selected articles encompass a wide array of types, including academic papers, conference proceedings, editorial materials, book reviews, and chapters. These documents collectively address diverse aspects of mindfulness, including therapy and intervention measures tailored to different populations, and explore various research directions such as the application of mindfulness in different therapeutic contexts and intervention strategies. After removing duplicates and irrelevant entries, a search conducted in December 2022 resulted in a corpus of 1,950 documents (Figure 1), forming the basis for subsequent bibliometric analyses.

The extracted data includes authors, paper titles, and keywords, which were inputted into the VOSviewer software for visual mapping. This software supports the comparison of normalized cluster networks, coverage visualization, and density visualization (Van Eck and Waltman, 2010), allowing for comprehensive bibliometric analysis through appropriately set threshold values.

## Results and discussion

### Yearly quantitative distribution of literature

**Figure 2:**
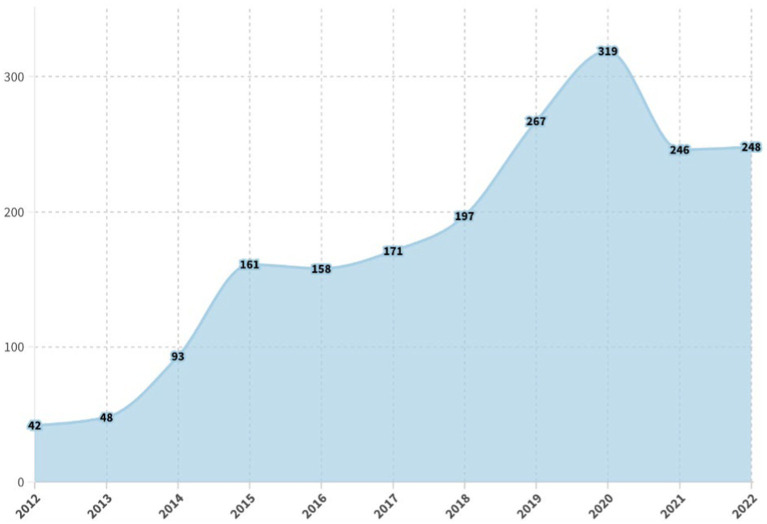
Yearly quantitative distribution of literature.

As shown in Figure 2, the journal *Mindfulness* has published a total of 1,950 articles in the WoS database as of December 2022. Since its inception in 2012, the annual publication volume has exhibited a consistent upward trajectory, delineated into three distinct stages: the “Emergence Stage” (2012–2014), where fewer than 100 articles were published each year; the “Exploration Stage” (2015–2018), characterized by a gradual increase in publication numbers, with 2015 marking the first year the journal exceeded 150 articles; and the “Growth Stage” (2019–2022), noted for a robust and stable trend of publishing over 200 articles annually starting in 2019. This latter stage underscores a burgeoning interest in mindfulness research. Nonetheless, there was a notable decline in publication numbers in 2021 and 2022, a trend likely influenced by the global disruptions caused by the COVID-19 pandemic.

### Significant publications in different development stages

Table 1 categorizes key literature from the journal *Mindfulness* into three developmental stages, highlighting the impact of these works through the lens of “the top three most-cited articles” in the WoS database. This method underscores the relevance and significance of these articles within their respective research domains.

**Table 1:** Significant publications in different development stages.

**Stage**	**Publication count**	**Most cited studies**	**Author/Year**	**Main findings**	**Citation count**
Emergence stage (2012—2014)	183	1	Eberth and Sedlmeier (2012)	Mindfulness meditation enhances emotional regulation, attention, and stress reduction.	402
		2	Meiklejohn et al. (2012)	Mindfulness training boosts self-awareness, emotional management, and learning in K-12 education.	309
		3	Sauer et al. (2013)	Recommendations for improving the accuracy of mindfulness measurement tools are proposed.	276
Exploration stage (2015—2018)	687	1	Neff (2016)	The Self-Compassion Scale is validated as an effective tool for measuring self-compassion.	347
		2	Zoogman et al. (2015)	Mindfulness meditation benefits for adolescents, particularly in clinical settings, are highlighted for future research.	344
		3	Tomlinson et al. (2018)	The positive link between trait mindfulness and psychological health is identified, with advice on addressing research limitations.	231
Growth stage (2019—2022)	1,080	1	Ferrari et al. (2019)	Self-compassion interventions are effective across various psychosocial outcomes, enhancing well-being and social skills.	187
		2	Flett et al. (2019)	Short-term mindfulness meditation improves psychological health, with potential long-term benefits.	105
		3	Wilson et al. (2019)	Various self-compassion therapies are effective for treating conditions like anxiety and depression, and they promote self-compassion and reduce psychopathology.	93

During the “Emergence Stage” (2012–2014), 183 articles were published. The top three most-cited articles included Eberth and Sedlmeier (2012), Meiklejohn et al. (2012), and Sauer et al. (2013). Eberth and Sedlmeier (2012) offered a comprehensive review of the effects of mindfulness meditation on various psychological variables among non-clinical meditators. Meiklejohn et al. (2012) explored the integration of mindfulness training into K-12 curricula, employing a combination of direct and indirect teaching methods facilitated by teacher training. This study highlighted that continuous mindfulness practice enhances attention and emotional regulation, benefiting both teachers and students. Sauer et al. (2013) emphasized the necessity of comparing mindfulness measurement results obtained through self-assessment tools with those from other mindfulness measurement tools, providing insights for improving current methodologies.

The “Exploration Stage” (2015–2018) produced 687 articles, with Zoogman et al. (2015), Neff (2016), and Tomlinson et al. (2018) being the most cited. Neff (2016) introduced the Self-Compassion Scale (SCS), establishing it as an effective measure of self-compassion and highlighting the importance of the “self-criticism” factor. Zoogman et al. (2015) investigated mindfulness-based interventions for adult depression and anxiety, suggesting potential applicability to adolescents, especially in non-clinical settings. Tomlinson et al. (2018) examined the correlation between trait mindfulness and mental health, indicating positive impacts and pointing out areas for future research, including addressing conceptual and methodological challenges in the field.

From 2019 to 2022, the “Growth Stage” saw the publication of 1,080 articles, with significant contributions from Ferrari et al. (2019), Flett et al. (2019), and Wilson et al. (2019). Ferrari et al. (2019) validated the effectiveness of self-compassion interventions in enhancing psychosocial outcomes. Flett et al. (2019) explored both the short-term and long-term benefits of mindfulness meditation on mental health. Wilson et al. (2019) reviewed therapies related to self-compassion, including compassion-focused therapy and mindfulness-based cognitive therapy, demonstrating significant improvements in conditions like anxiety and depression, thus promoting self-compassion and reducing psychopathology among both clinical and subclinical populations.

In summary, each developmental stage of *Mindfulness* research progressively explores different facets, with a significant emphasis on the management and regulation of psychological processes like self-regulation, emotions, and psychological health, which are increasingly recognized as central themes in contemporary mindfulness research.

### Distribution of authors

**Figure 3:**
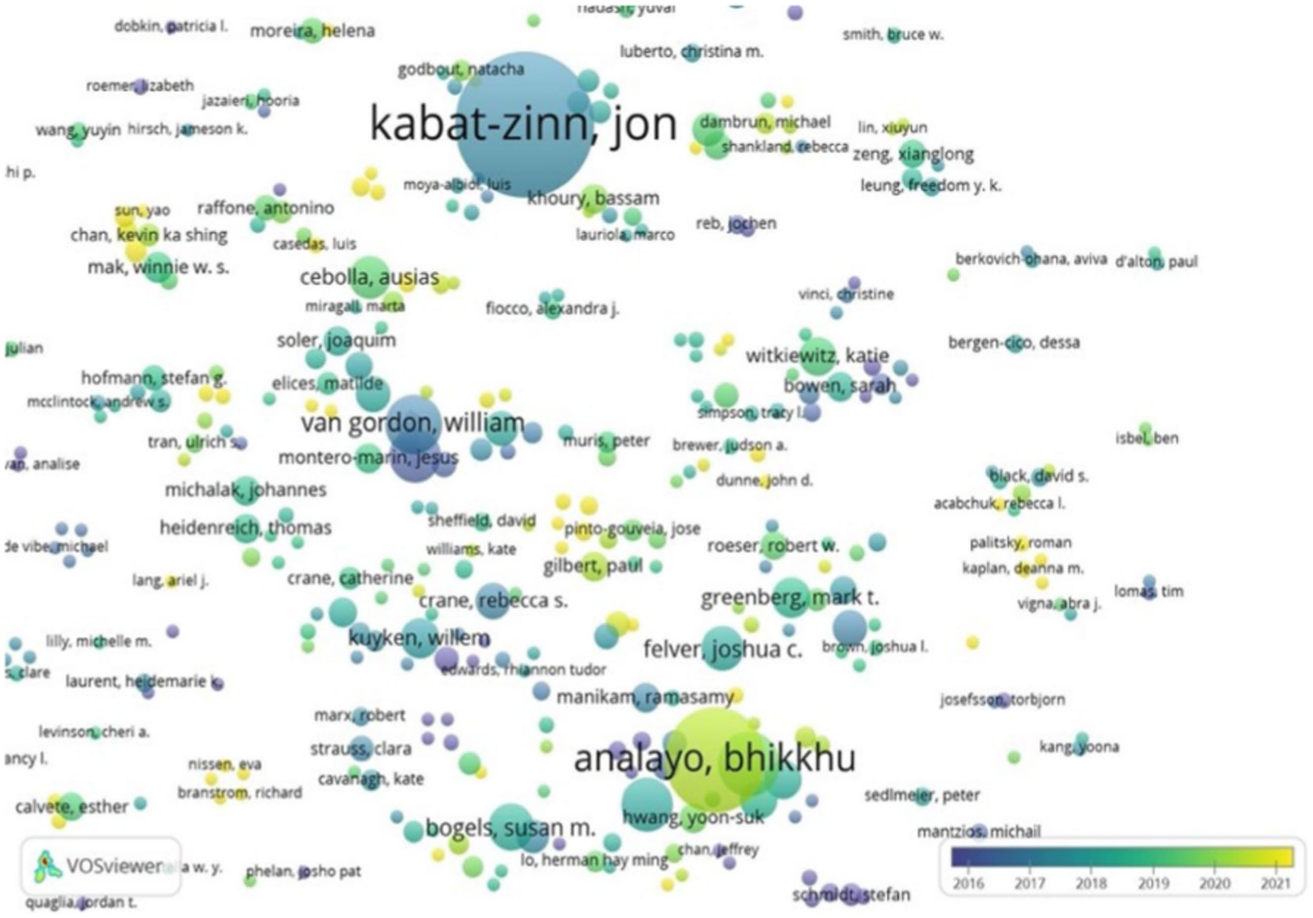
Distribution of authors.

As shown in Figure 3, this study’s analysis of author distribution provides insights into their connections with international scholars. Among the 200 authors featured on Mindfulness, notable contributors include Kabat-Zinn, Analayo, Van Gordon, Medvedev and Bögels.

In 1979, Dr. Kabat-Zinn launched the MBSR program at the University of Massachusetts Medical School, effectively helping patients handle stress, pain, and illness through mindfulness techniques. His method, practiced in over 200 medical institutions across North America, has significantly influenced healthcare, education, and other sectors for decades. Dr. Kabat-Zinn’s numerous publications, including *Full Catastrophe Living and The Mindful Way Through Depression,* have further popularized these approaches (Kabat-Zinn, 2023).

Dr. Analayo of the Barre Center for Buddhist Studies and the Numata Centre for Buddhist Studies at The University of Hamburg focuses on early Buddhist texts and meditation practices. His work bridges ancient Buddhist techniques with modern practices, exploring mindfulness as a connection between mind and body, vital for continuous awareness in daily life (Anālayo, 2020).

Dr. Van Gordon, from the University of Derby, has established credibility in studying the efficacy of Buddhist-derived meditations like Loving-Kindness Meditation (LKM) and Compassion Meditation (CM) in treating a range of mental health problems. His research emphasizes the foundational importance of Meditation Awareness Training (MAT) in enhancing psychological well-being in educational settings among other applications (University of Derby, 2023).

Dr. Medvedev from the University of Waikato has refined the Five Facet Mindfulness Questionnaire using Rasch analysis to enhance its precision and validity, supporting its application in diverse psychological and health-related fields (The University of Waikato, 2023). His research covers various fields, such as assessment methods, health psychology, psychophysiology, and biostatistics.

Dr. Bögels, a professor at the University of Amsterdam, has extensively researched the interplay between cognitive-behavioral therapy and mood disorders in treating childhood social anxiety (University of Amsterdam, 2023). Her findings on the effectiveness of mindful parenting as a therapeutic intervention highlight its benefits in reducing stress and improving family dynamics (Bögels et al., 2014).

As shown in Table 2, Kabat-Zinn, Analayo, and Van Gordon have predominantly focused on exploring aspects of Buddha’s teachings, the inherent purity of the meditator’s mind, and Meditation Awareness Mindfulness, among other elements. Analayo and Van Gordon bring unique perspectives to their empirical research on meditation’s role in regulating personal physical and mental states, enhancing internal awareness, insight, compassion, and peace. Kabat-Zinn, on the other hand, has been pivotal in integrating mindfulness into psychological therapy and neuropsychology, significantly advancing the therapeutic landscape by mitigating physical and mental distress and promoting overall well-being. Their collective research emphasizes the efficacy of mindfulness interventions in alleviating anxiety and stress, while also advocating for the enhancement of physical and mental health and overall happiness. Medvedev, renowned for his expertise in assessing mindfulness, excels in documenting the observable benefits and self-regulation strategies of mindfulness training through the use of questionnaires, observations, and interviews. In contrast, Bögels concentrates on the application of mindfulness counseling treatment to address stress, depressive mood, and situational trait anxiety among children and their parents, revealing significant benefits in children’s cognition, social interaction, self-care, and mental health.

**Table 2:** Distribution of authors.

**Most published authors**	**Author**	**Affiliated institution**	**Country**	**Main research areas**	**Publication count**
1	Kabat-Zinn	University of Massachusetts System	USA	Focuses on integrating Buddhist mindfulness meditation training into medical practice and is the founder of MBSR.	116
2	Analayo	Barre Center For Buddhist Studies/ Numata Centre For Buddhist Studies At The University Of Hamburg	USA/ Germany	Concentrates on early Buddhist scriptures, the theory and practice of meditation, and explores the historical development of mindfulness and Buddhist thought.	70
3	Van Gordon	University Of Derby	U.K.	Founded Meditation Awareness Training and researches the applications of mindfulness meditation training.	29
4	Medvedev	University Of Waikato	New Zealand	Focuses on mindfulness interventions, psychopathology, health, and well-being, along with the study of mindfulness assessment techniques.	28
5	Bögels	University of Amsterdam	Netherlands	Focuses on diagnostics and applications in mental health, researching mental health treatments for children and parents.	25

### Distribution of countries and institutions

**Figure 4:**
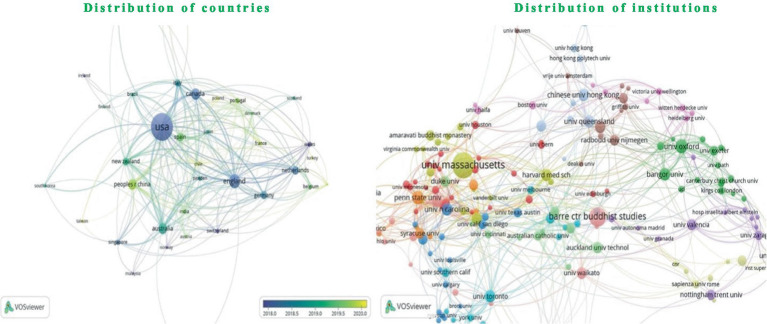
Distribution of countries and institutions.

As shown in Figure 4, this study analyzes the distribution of publications and institutions to elucidate geographical knowledge networks within the field of mindfulness. An examination of publications from the *Mindfulness* journal indicates a wide international spread, involving researchers from 68 countries, with the United States, United Kingdom, Canada, Australia, and China being the primary contributors. Among these, significant institutions include the University of Massachusetts System, University of Massachusetts Worcester, Barre Center for Buddhist Studies, University of California System, and University System of Georgia, all located in the USA, underscoring the predominant role of the United States in mindfulness research. Notably, the focus of Chinese research is primarily centered in Hong Kong, signaling its prominence in China’s mindfulness studies, while suggesting that other regions in China could enhance their contributions to this field.

As shown in Table 3, the United States leads in publication volume, followed by the United Kingdom, with substantial inputs from Canada and Australia, whereas China exhibits fewer publications. This distribution underscores a pronounced interest and earlier initiation of mindfulness research among scholars in the US and UK. Institutions like the University of Massachusetts System, Barre Center for Buddhist Studies, and University of Derby, which house principal authors in mindfulness research, are closely aligned with core fields such as mindfulness meditation, training, measurement, intervention, and regulation. This alignment reflects a concentrated and specialized focus in the developmental stages of mindfulness research.

**Table 3:** Distribution of countries and institutions.

**Most published countries and institutions**	**Country**	**Institution**	**Main research areas**	**Publication count**
1	USA	University of Massachusetts System	Researches the origins of mindfulness and its application in psychopathology.	114
		University of Massachusetts Worcester	Explores the connection between mindfulness and Buddhism and the application of mindfulness interventions and stress reduction.	105
		Barre CTR Buddhist Studies	Studies mindfulness-related meditation and the positioning of mindfulness in early Buddhism.	66
		University of California System	Investigates the application of mindfulness in emotional regulation therapy and its effects on stress reduction.	53
		University System of Georgia	Examines the effects of mindfulness therapy on adolescents and parenting and its role in emotional regulation.	45
2	U.K.	University of Oxford	Researches the effects of mindfulness in reducing stress and its derivative therapies and cognitive therapy of mindfulness intervention.	34
		University of London	Measures mindfulness in children and adolescents and studies mindfulness interventions and cognitive therapy.	28
		University of Derby	Explores mindfulness and traditional meditation practices and assesses the impact of mindfulness on psychophysical health.	27
		Nottingham Trent University	Develops mindfulness measurement scales and practices mindfulness meditation.	21
		Amaravati Buddhist Monastery	Explores the origins of mindfulness in classical Buddhism.	19
3	Canada	University of Toronto	Studies the effects of mindfulness in regulating children’s mental health and emotional regulation.	32
		Mcgill University	Researches mindfulness meditation and mindfulness intervention therapy.	21
		University of Western Ontario	Explores the application and treatment of mindfulness in clinical psychology.	16
		University of British Columbia	Uses mindfulness measurement scales in psychiatry and mindfulness intervention therapy.	13
		University of Quebec	Measures mindfulness and emotion regulation therapy in children and adolescents.	12
4	Australia	University of Queensland	Researches various interventions of mindfulness.	29
		Monash University	Studies mindfulness training in the workplace and mindfulness interventions in special groups.	20
		Australian Catholic University	Researches the effects of mindfulness in reducing stress and the connection between mindfulness and happiness.	17
		University of Melbourne	Practices and studies the effects of mindfulness meditation.	12
		University of Western Australia	Explores the connection of mindfulness with self-compassion, self-regulation, and self-kindness and its effects.	12
5	China	Chinese University of Hong Kong	Studies the interventions and effects of mindfulness.	28
		Beijing Normal University	Examines the measures and effects of mindfulness in emotional regulation.	22
		Education University of Hong Kong	Researches the effect of mindfulness in parenting and children’s self-regulation.	21
		University of Hong Kong	Studies the effects of mindfulness in the regulation of mental health of adolescents.	13
		Hong Kong Polytechnic University	Researches mindfulness interventions in children and parents.	10

### Keyword co-occurrence

**Figure 5:**
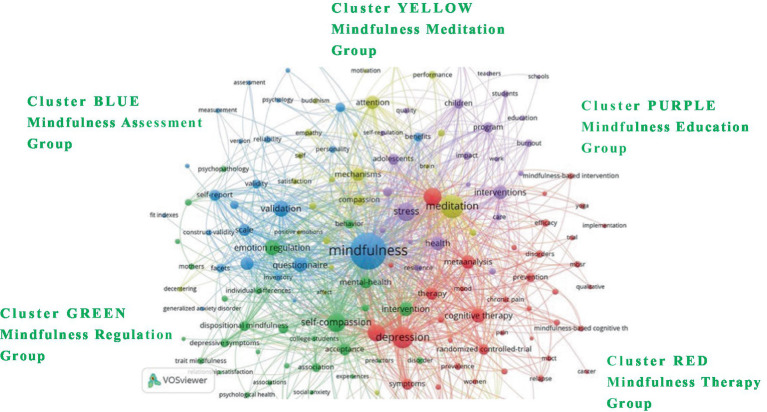
Keyword co-occurrence.

The analysis of keyword co-occurrence in this study is based on the size of network nodes, which represents the importance of each keyword. The larger the keyword, the closer it is to the research hotspot. As shown in Figure 5, the high-frequency keywords in the *Mindfulness* journal are ‘mindfulness,’ ‘meditation,’ ‘depression,’ ‘self-compassion,’ and ‘stress,’ all of which are at the core of the clusters.

As shown in Table 4, “Mindfulness” is identified as the central term across all articles, reflecting its prevalent usage within the field. The analysis reveals other significant keywords such as “meditation,” “depression,” “self-compassion,” and “stress.” Notably, “meditation” was a dominant theme in the initial stages of research, with a marked increase in related studies between 2014 and 2018, while “self-compassion” gained prominence around 2020. This study organizes these keywords into five distinct clusters based on node size. The red cluster, focusing on “depression,” incorporates themes like anxiety, systematic analysis, cognitive therapy, and treatment, primarily concerning mindfulness treatment. The green cluster, centered around “self-compassion,” includes terms related to emotion regulation, intervention, psychological health, and acceptance, highlighting aspects of mindfulness regulation. The blue cluster, led by “mindfulness,” deals with the facets of examination, questionnaires, psychometric properties, and grading, pertinent to mindfulness assessment. The yellow cluster, under the banner of “meditation,” delves into mechanisms, attention, compassion, and empathy, enriching the discourse on mindfulness meditation. Lastly, the purple cluster, themed around “stress,” addresses issues related to health, adolescents, well-being, and education, underscoring mindfulness education. Collectively, these clusters illustrate the breadth of mindfulness research, showcasing a range of topics from treatment and regulation to assessment and educational applications, reflecting the evolving dynamics and the comprehensive scope of mindfulness as a research field.

**Table 4:** Keyword co-occurrence analysis.

**Cluster**	**Count**	**Focus**	**High-frequency keywords**	**Frequency**	**Link strength**
Cluster 1. Red [Mindfulness Therapy Group]	42	Focuses on treating anxiety and negative emotions using cognitive therapy and systematic analysis within mindfulness therapy.	Depression	411	3,072
Cluster 2. Green [Mindfulness Regulation Group]	38	Targets enhancing psychological health through emotional regulation and acceptance interventions in mindfulness.	Self-Compassion	306	2,177
Cluster 3. Blue [Mindfulness Assessment Group]	25	Develops and refines questionnaires to accurately assess the psychometric properties of mindfulness.	Mindfulness	1,109	7,148
Cluster 4. Yellow [Mindfulness Meditation Group]	24	Investigates how mindfulness meditation enhances attention, compassion, and empathy to improve interpersonal understanding.	Meditation	482	3,195
Cluster 5. Purple [Mindfulness Education Group]	24	Promotes health and well-being in adolescents by incorporating mindfulness into psychophysical educational programs.	Stress	298	2075

The interconnected themes highlighted by these keywords underscore the varied research focus directions of the journal *Mindfulness* in recent years, reflecting the dynamic evolution of paradigms within mindfulness research. This body of work integrates several core areas, including addressing unhealthy, negative, and adverse emotions through mindfulness-based interventions, exploring strategies for mental health and emotion regulation, conducting evaluations with mindfulness-related questionnaires, investigating meditation practices to foster understanding and empathy, and developing psychophysical educational programs specifically designed for adolescents. Collectively, these focal points illustrate the journal’s commitment to advancing both the theoretical and practical aspects of mindfulness, contributing significantly to our understanding of its diverse applications across various contexts.

### Reference co-citation

**Figure 6:**
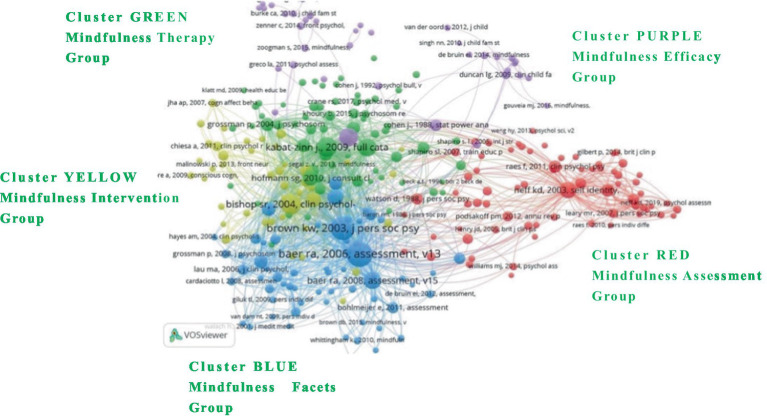
Reference co-citation.

This study aims to explore the development and dynamic evolution of themes and their relationships within the mindfulness research field, thereby enhancing our understanding of its current state and providing valuable scientific guidance for scholars. As shown in Figure 6, an analysis of references from the *Mindfulness* journal reveals that reference co-citations are divided into five clusters: the red cluster focuses on mindfulness assessment with 70 articles, primarily exploring the development and validation of related scales; the green cluster, comprising 65 articles, assesses various mindfulness therapies; the blue cluster, with 62 articles, discusses the structural aspects of mindfulness; the yellow cluster includes 43 articles on mindfulness intervention, evaluating its structural composition and clinical intervention mechanisms; and the purple cluster, consisting of 37 articles, measures the effectiveness of mindfulness across medicine, psychology, education, and other fields. Prominent researchers contributing to these clusters include Kabat-Zinn (2003), Neff (2003a), Bishop et al. (2004), Baer et al. (2006), and Kabat-Zinn (2009), whose works significantly shape the discourse within these areas.

As shown in Table 5, the article “Using Self-Report Assessment Methods to Explore Facets of Mindfulness” by Baer et al. (2006) stands out as the most strongly linked article, published by the American Psychological Association, Society for Clinical Psychology (Division 12), Section IX (Assessment). This pivotal article investigates various methods and approaches for self-assessing mindfulness. Following closely is “The Benefits of Being Present: Mindfulness and Its Role in Psychological Well-Being” by Brown and Ryan (2003), which utilized the Mindful Attention Awareness Scale (MAAS) to analyze mindfulness’s predictive and regulatory role in psychological health, published in Personality and Social Psychology. The reference co-citations related to these articles are organized into five clusters that reflect their influence and connections within the field. The red cluster, focusing on mindfulness assessment, is highlighted by Baer et al. (2006), who discuss the multifaceted nature of mindfulness and its assessment techniques. This cluster emphasizes articles that delve into the development and validation of scales designed to measure mindfulness attributes accurately. The green cluster centers around mindfulness therapy, featuring Hofmann et al. (2010) who confirm the effectiveness of mindfulness therapies in treating clinical issues like anxiety and depression. This cluster collectively examines the therapeutic applications and outcomes of mindfulness-based interventions. In the blue cluster, which addresses the structural aspects of mindfulness, Brown and Ryan (2003) explore the role of mindfulness in enhancing psychological well-being, showcasing its regulatory impact on mental health through empirical studies. The yellow cluster, dedicated to mindfulness interventions, includes Kabat-Zinn (2009), whose work discusses practical mindfulness applications in dealing with stress, pain, and illness, emphasizing the operational mechanisms and clinical efficacy of mindfulness. Finally, the purple cluster, focusing on the effectiveness of mindfulness, features Neff (2003a) who develops and validates the Self-Compassion Scale, exploring the beneficial effects of self-compassion as part of a mindfulness approach. This cluster explores how mindfulness practices contribute to overall health and education, highlighting their potential in fostering enhanced well-being across various populations. These clusters demonstrate the journal *Mindfulness*’s comprehensive coverage of research that spans theoretical explorations to practical applications, reflecting the dynamic and evolving landscape of mindfulness research.

**Table 5:** Reference co-citation analysis.

**Cluster**	**Count**	**Focus**	**High-frequency co-cited reference (Author/Year)**	**Main findings**	**Link strength**
Cluster 1. Red [Mindfulness Assessment Group]	70	Development and validation of related scales	Neff (2003a)	Developed and validated the Self-Compassion Scale to measure self-compassion accurately.	3,376
			Neff (2003b)	Explored the link between self-compassion and psychological functioning, identifying potential differences across groups.	1999
			Raes et al. (2011)	Constructed and validated a short version of the Self-Compassion Scale to facilitate easier assessment of self-compassion.	1772
Cluster 2. Green [Mindfulness Therapy Group]	65	Mindfulness therapy	Kabat-Zinn (2009)	Described using the inherent wisdom of body and mind to manage stress, pain, and illness effectively.	4,214
			Segal and Ferguson (2018)	Outlined the principles and effectiveness of mindfulness-based cognitive therapy.	4,078
			Hofmann et al. (2010)	Confirmed the efficacy of mindfulness therapy as a promising intervention for treating clinical anxiety and emotional disturbances.	3,357
Cluster 3. Blue [Mindfulness Facets Group]	62	Mindfulness structural aspects	Baer et al. (2006)	Conceptualized mindfulness, helping to understand its components and their connections to various psychological variables.	7,718
			Brown and Ryan (2003)	Discussed how mindfulness positively affects psychological health.	7,203
			Baer et al. (2008)	Confirmed the validity of the Five Facet Mindfulness Questionnaire, demonstrating that mindfulness can be effectively measured with the FFMQ.	3,958
Cluster 4. Yellow [Mindfulness Intervention Group]	43	Structure and clinical intervention mechanisms of mindfulness	Bishop et al. (2004)	Addressed conceptual and operational issues in mindfulness, enhancing understanding of its clinical applications.	5,245
			Shapiro et al. (2006)	Outlined the positive effects of mindfulness and discussed the outcomes and future directions of mindfulness interventions for psychological and physical symptoms.	3,006
			Hölzel et al. (2011)	Proposed mechanisms of action for mindfulness meditation and discussed its operational components.	2,333
Cluster 5. Purple [Mindfulness Efficacy Group]	37	Effectiveness of mindfulness interventions in various domains such as medicine, psychology, and education	Kabat-Zinn (2003)	Discussed the effectiveness of mindfulness interventions in clinical settings, underscoring their therapeutic potential.	3,864
			Cohen (1988)	Analyzed the importance of statistical power in behavioral science research.	1,566
			Duncan et al. (2009)	Discussed mindfulness-based parenting interventions, emphasizing their role in fostering emotional awareness and self-regulation.	803

## Conclusion and implications

This study employs bibliometric analysis to conduct a visual analysis of research published in the *Mindfulness* journal, aiming to provide scholars with a relatively objective perspective to grasp the dynamics and future directions of international mindfulness research. The findings indicate that over the past decade, the journal has published 1,950 articles, with publication numbers increasing over time. Among the many contributors, Kabat-Zinn, Analayo, Van Gordon, Medvedev, and Bögels stand out as key figures, with mindfulness research predominantly concentrated in Western countries, particularly the United States and the United Kingdom, which have had the most significant impact on the field. The research primarily focuses on themes such as mindfulness, meditation, depression, stress, and self-compassion. Moreover, the studies are extensively centered around specific aspects of mindfulness, including “intervention,” “therapy,” “regulation,” “assessment,” and “education.” In Taiwan, mindfulness research is relatively underdeveloped; the analysis of this data not only helps identify current research hotspots and gaps but also provides valuable references for researchers in Taiwan, further facilitating the extensive application and in-depth development of mindfulness studies.

We conducted a systematic analysis of various dimensions within mindfulness research, including institutions, nations, individual researchers, and trending topics, thus uncovering key interconnections among these elements. The distribution of these relationships not only maps the trajectory of mindfulness research but also highlights the global imbalance in research capabilities. Particularly, the cultural drivers and relationships between research hotspots, regions, institutions, and individual researchers are crucial as they facilitate collaboration across geographical, disciplinary, and cultural boundaries, which is vital for the global application and dissemination of mindfulness. For instance, Hofmann et al. (2010) have confirmed through their comprehensive analysis that mindfulness therapy positively impacts symptoms of anxiety and depression, a finding that is consistently underscored by frequent references to “depression” and “therapy.” The high-frequency keywords and reference co-citations exhibit a robust linkage pattern, illustrating interrelated connections among these themes. Not only do these connections enrich the existing literature, but they also provide invaluable references for the further development of mindfulness research, highlighting its significance across various psychological and educational settings. Although Asian countries have lesser participation in mindfulness research, their rich history of traditional meditation practices offers substantial untapped potential for future studies. Strengthening collaborations with Western countries can enhance the exchange of knowledge and technologies, bringing fresh perspectives that are essential for advancing the globalization of mindfulness research.

### Implications for mindfulness research and education in Taiwan

In Taiwan, mindfulness research is still in its nascent stages, with a notable absence of publications in the international journal *Mindfulness*, indicative of a lack of systematic research. Scholars in Taiwan are thus encouraged to align with international research trends in mindfulness, enhancing their analytical approaches. There is a strong recommendation for scholars to focus more on demographic groups that could benefit from improved mental health. This involves intensifying global dialogue and exchange between domestic scholars and their international counterparts, which is vital for understanding the structural and developmental nuances of mindfulness research. This approach will facilitate international comparative studies, promote scientific collaboration globally, and provide robust support for individuals in high-pressure work environments. To improve Taiwan’s education directions and aid Taiwanese researchers in thoroughly exploring the development status and trends of the international mindfulness research fields, this study proposes several strategies to accelerate the internationalization of domestic research and discipline construction. These include integrating mindfulness education into curricula at all educational levels to provide students with systematic training in mindfulness practices like meditation, emotion regulation, and concentration; developing mindfulness teacher training programs to enhance educators’ emotional management skills; promoting mindfulness-friendly campuses to foster a respectful, caring, and harmonious learning environment; integrating mindfulness into special education as an auxiliary therapy for students with conditions such as autism and ADHD to enhance their emotion regulation and self-control; and conducting thorough research and assessments of mindfulness education to gauge its impact on students’ learning outcomes, psychological health, and interpersonal relationships, thereby generating empirical evidence to support the expansion of mindfulness education.

### Limitations, and suggestions for future research

While this study presents notable findings, it is not without its limitations. The analysis relies exclusively on literature data from the Journal of *Mindfulness* in the WoS database, lacking empirical field investigations and experimental validation. This focus restricts the breadth of mindfulness-related literature reviewed, as it does not consider contributions from other journals. Future research could benefit from employing a variety of research methods and data sources, integrating themes such as “intervention, therapy, regulation, assessment, education” to expand the exploration of mindfulness applications across diverse domains and populations. Additionally, by prioritizing key terms within the co-occurrence patterns, new research avenues can be identified, which will drive the development of mindfulness research and offer valuable guidance for practical applications and policy formulation.

## Data availability statement

The original contributions presented in the study are included in the article/supplementary material, further inquiries can be directed to the corresponding author.

## Author contributions

C-CH: Writing – review & editing, Supervision. SL: Writing – original draft, Writing – review & editing.
